# Supplementary material for: Allosteric regulation and crystallographic fragment screening of SARS-CoV-2 NSP15 endoribonuclease
Source: Nucleic Acids Res. 2023 Apr 28;51(10):5255–70. doi: 10.1093/nar/gkad314 (PMC10250223; doi:10.1093/nar/gkad314)
Supplement: gkad314_Supplemental_Files [file gkad314_supplemental_files.zip › Supplementary datasets.zip!/159/Refine_4/01-BUSTER/Cycle-3/shell.01/R_Rfree.html]

# Rxpct factors in resolution bins at cycle 0


|  |  |  |  |  |  |  |  |  |
| --- | --- | --- | --- | --- | --- | --- | --- | --- |
|  | **<d> (Å)**  Rxpct\_Work | Rxpct\_Free | Rxpct\_All | |  |  |  |  |
|  | 12.8357 | 0.3170 | 0.4072 | 0.3208 |  |
|  | 7.4107 | 0.2654 | 0.2864 | 0.2667 |  |
|  | 5.7403 | 0.2690 | 0.2982 | 0.2701 |  |
|  | 4.8514 | 0.2092 | 0.2341 | 0.2106 |  |
|  | 4.2786 | 0.2051 | 0.1969 | 0.2047 |  |
|  | 3.8701 | 0.2141 | 0.2376 | 0.2153 |  |
|  | 3.5600 | 0.2282 | 0.2603 | 0.2297 |  |
|  | 3.3142 | 0.2333 | 0.2720 | 0.2357 |  |
|  | 3.1131 | 0.2293 | 0.2391 | 0.2298 |  |
|  | 2.9447 | 0.2159 | 0.2233 | 0.2163 |  |
|  | 2.8010 | 0.2102 | 0.2153 | 0.2105 |  |
|  | 2.6764 | 0.2014 | 0.2167 | 0.2022 |  |
|  | 2.5671 | 0.1942 | 0.1988 | 0.1945 |  |
|  | 2.4702 | 0.1883 | 0.1939 | 0.1886 |  |
|  | 2.3835 | 0.1854 | 0.2002 | 0.1861 |  |
|  | 2.3054 | 0.1769 | 0.1951 | 0.1778 |  |
|  | 2.2344 | 0.1881 | 0.2043 | 0.1888 |  |
|  | 2.1696 | 0.1922 | 0.1974 | 0.1925 |  |
|  | 2.1102 | 0.2021 | 0.2061 | 0.2023 |  |
|  | 2.0554 | 0.2265 | 0.2157 | 0.2260 |  |
|  | **Overall** | 0.2218 | 0.2401 | 0.2227 |  |

# Rxpct factors in resolution bins at cycle 1


|  |  |  |  |  |  |  |  |  |
| --- | --- | --- | --- | --- | --- | --- | --- | --- |
|  | **<d> (Å)**  Rxpct\_Work | Rxpct\_Free | Rxpct\_All | |  |  |  |  |
|  | 12.8357 | 0.3238 | 0.4137 | 0.3275 |  |
|  | 7.4107 | 0.2714 | 0.2928 | 0.2727 |  |
|  | 5.7403 | 0.2707 | 0.3011 | 0.2719 |  |
|  | 4.8514 | 0.2086 | 0.2343 | 0.2100 |  |
|  | 4.2786 | 0.2031 | 0.1955 | 0.2027 |  |
|  | 3.8701 | 0.2115 | 0.2371 | 0.2127 |  |
|  | 3.5600 | 0.2270 | 0.2581 | 0.2285 |  |
|  | 3.3142 | 0.2309 | 0.2719 | 0.2334 |  |
|  | 3.1131 | 0.2278 | 0.2361 | 0.2282 |  |
|  | 2.9447 | 0.2142 | 0.2203 | 0.2144 |  |
|  | 2.8010 | 0.2089 | 0.2143 | 0.2092 |  |
|  | 2.6764 | 0.2014 | 0.2169 | 0.2023 |  |
|  | 2.5671 | 0.1947 | 0.2004 | 0.1951 |  |
|  | 2.4702 | 0.1892 | 0.1949 | 0.1895 |  |
|  | 2.3835 | 0.1864 | 0.2009 | 0.1871 |  |
|  | 2.3054 | 0.1789 | 0.1988 | 0.1798 |  |
|  | 2.2344 | 0.1893 | 0.2034 | 0.1900 |  |
|  | 2.1696 | 0.1939 | 0.1996 | 0.1942 |  |
|  | 2.1102 | 0.2035 | 0.2087 | 0.2038 |  |
|  | 2.0554 | 0.2288 | 0.2173 | 0.2282 |  |
|  | **Overall** | 0.2218 | 0.2405 | 0.2227 |  |

# Rxpct factors in resolution bins at cycle 2


|  |  |  |  |  |  |  |  |  |
| --- | --- | --- | --- | --- | --- | --- | --- | --- |
|  | **<d> (Å)**  Rxpct\_Work | Rxpct\_Free | Rxpct\_All | |  |  |  |  |
|  | 12.8357 | 0.3204 | 0.4085 | 0.3241 |  |
|  | 7.4107 | 0.2677 | 0.2902 | 0.2691 |  |
|  | 5.7403 | 0.2674 | 0.2989 | 0.2686 |  |
|  | 4.8514 | 0.2073 | 0.2316 | 0.2086 |  |
|  | 4.2786 | 0.2033 | 0.1955 | 0.2028 |  |
|  | 3.8701 | 0.2123 | 0.2367 | 0.2135 |  |
|  | 3.5600 | 0.2275 | 0.2572 | 0.2290 |  |
|  | 3.3142 | 0.2314 | 0.2737 | 0.2340 |  |
|  | 3.1131 | 0.2276 | 0.2349 | 0.2279 |  |
|  | 2.9447 | 0.2137 | 0.2204 | 0.2140 |  |
|  | 2.8010 | 0.2083 | 0.2118 | 0.2085 |  |
|  | 2.6764 | 0.2006 | 0.2151 | 0.2014 |  |
|  | 2.5671 | 0.1936 | 0.1997 | 0.1940 |  |
|  | 2.4702 | 0.1877 | 0.1924 | 0.1879 |  |
|  | 2.3835 | 0.1856 | 0.2017 | 0.1864 |  |
|  | 2.3054 | 0.1768 | 0.1964 | 0.1777 |  |
|  | 2.2344 | 0.1886 | 0.2041 | 0.1893 |  |
|  | 2.1696 | 0.1930 | 0.1993 | 0.1933 |  |
|  | 2.1102 | 0.2032 | 0.2067 | 0.2033 |  |
|  | 2.0554 | 0.2292 | 0.2148 | 0.2284 |  |
|  | **Overall** | 0.2210 | 0.2394 | 0.2219 |  |

# Rxpct factors in resolution bins at cycle 3


|  |  |  |  |  |  |  |  |  |
| --- | --- | --- | --- | --- | --- | --- | --- | --- |
|  | **<d> (Å)**  Rxpct\_Work | Rxpct\_Free | Rxpct\_All | |  |  |  |  |
|  | 12.8357 | 0.3229 | 0.4111 | 0.3266 |  |
|  | 7.4107 | 0.2675 | 0.2923 | 0.2690 |  |
|  | 5.7403 | 0.2635 | 0.2957 | 0.2648 |  |
|  | 4.8514 | 0.2055 | 0.2343 | 0.2071 |  |
|  | 4.2786 | 0.2018 | 0.1990 | 0.2016 |  |
|  | 3.8701 | 0.2116 | 0.2405 | 0.2130 |  |
|  | 3.5600 | 0.2265 | 0.2535 | 0.2278 |  |
|  | 3.3142 | 0.2302 | 0.2734 | 0.2328 |  |
|  | 3.1131 | 0.2251 | 0.2392 | 0.2257 |  |
|  | 2.9447 | 0.2127 | 0.2175 | 0.2129 |  |
|  | 2.8010 | 0.2058 | 0.2103 | 0.2060 |  |
|  | 2.6764 | 0.1983 | 0.2098 | 0.1990 |  |
|  | 2.5671 | 0.1915 | 0.1980 | 0.1919 |  |
|  | 2.4702 | 0.1846 | 0.1890 | 0.1848 |  |
|  | 2.3835 | 0.1826 | 0.1988 | 0.1834 |  |
|  | 2.3054 | 0.1722 | 0.1928 | 0.1731 |  |
|  | 2.2344 | 0.1855 | 0.1996 | 0.1861 |  |
|  | 2.1696 | 0.1886 | 0.1970 | 0.1891 |  |
|  | 2.1102 | 0.1994 | 0.1978 | 0.1993 |  |
|  | 2.0554 | 0.2234 | 0.2128 | 0.2229 |  |
|  | **Overall** | 0.2192 | 0.2392 | 0.2202 |  |
